# Identification and Characterization of Antifungal Compounds Using a *Saccharomyces cerevisiae* Reporter Bioassay

**DOI:** 10.1371/journal.pone.0036021

**Published:** 2012-05-04

**Authors:** Brad Tebbets, Douglas Stewart, Stephanie Lawry, Jeniel Nett, Andre Nantel, David Andes, Bruce S. Klein

**Affiliations:** 1 The Department of Pediatrics, University of Wisconsin School of Medicine and Public Health, Madison, Wisconsin, United States of America; 2 Department of Medicine, University of Wisconsin School of Medicine and Public Health, Madison, Wisconsin, United States of America; 3 Department of Internal Medicine, University of Wisconsin School of Medicine and Public Health, Madison, Wisconsin, United States of America; 4 Department of Medical Microbiology and Immunology, University of Wisconsin School of Medicine and Public Health, Madison, Wisconsin, United States of America; 5 The Cellular and Molecular Pathology Program, The University of Wisconsin-Madison, Madison, Wisconsin, United States of America; 6 Microbiology Doctoral Training Program, The University of Wisconsin-Madison, Madison, Wisconsin, United States of America; 7 Biotechnology Research Institute, The National Research Council of Canada, Montreal, Quebec, Canada; Montana State University, United States of America

## Abstract

New antifungal drugs are urgently needed due to the currently limited selection, the emergence of drug resistance, and the toxicity of several commonly used drugs. To identify drug leads, we screened small molecules using a *Saccharomyces cerevisiae* reporter bioassay in which *S. cerevisiae* heterologously expresses Hik1, a group III hybrid histidine kinase (HHK) from *Magnaporthe grisea*. Group III HHKs are integral in fungal cell physiology, and highly conserved throughout this kingdom; they are absent in mammals, making them an attractive drug target. Our screen identified compounds 13 and 33, which showed robust activity against numerous fungal genera including *Candida* spp., *Cryptococcus* spp. and molds such as *Aspergillus fumigatus* and *Rhizopus oryzae*. Drug-resistant *Candida albicans* from patients were also highly susceptible to compounds 13 and 33. While the compounds do not act directly on HHKs, microarray analysis showed that compound 13 induced transcripts associated with oxidative stress, and compound 33, transcripts linked with heavy metal stress. Both compounds were highly active against *C. albicans* biofilm, *in vitro* and *in vivo*, and exerted synergy with fluconazole, which was inactive alone. Thus, we identified potent, broad-spectrum antifungal drug leads from a small molecule screen using a high-throughput, *S. cerevisiae* reporter bioassay.

## Introduction

Over the past twenty years, the incidence of fungal infections has risen sharply as advances in medicine have increased the number of immunocompromised patients [1]. Unfortunately, the antifungal drug armamentarium has not kept pace. Current antifungal therapeutics are plagued with problems including limited spectrum of activity, the emergence of resistant strains, and patient toxicity [2]. New drugs are required to meet the growing need for antifungal therapy.

The identification of novel antifungals is hindered by the limited number of drug targets that are unique to fungi due to the close evolutionary relationship between fungi and mammals. Hybrid histidine kinases (HHKs) are an appealing antifungal drug target due to their central role in fungal physiology, conservation throughout the fungal kingdom, and absence in mammals. HHKs regulate two-component signaling pathways in response to a variety of environmental stimuli, including osmotic, nitrosative, and oxidative and stress in bacteria and fungi [3].

Two-component signal transduction cascades contain a sensor kinase and a response regulator. The sensor kinase regulates the pathway via phosphotransfer, where the kinase autophosphorylates a histidine residue and then transfers the phosphate to an aspartate on the response regulator. A hybrid histidine kinase contains both a kinase and a response regulator domain. Analysis of several fungal genomes has revealed 11 distinct HHK groups based on phylogenetic analysis of protein sequence [4]. Among these groups, the group III HHKs are the most attractive drug target due to their diverse regulon, which includes pleotropic phenotypes such as morphogenesis, virulence factor expression, and cell wall biogenesis. Group III HHKs contribute to virulence in the two most common systemic human fungal pathogens, *Aspergillus fumigatus* [5] and *Candida albicans* [6], and they regulate the phase transition, sporulation, and virulence factor expression in the dimorphic fungal pathogens *Blastomyces dermatitidis* and *Histoplasma capsulatum* [3], [7].

Group III HHKs in *C. albicans*, *A. fumigatus*, and *Magnaporthe grisea* are a target of the agricultural antifungal compound fludioxonil. Deletion of the group III HHK in these fungi renders them resistant to fludioxonil [8]–[10]. Conversely, heterologous expression of the group III HHK, Hik1, from *M. grisea* in *S. cerevisiae* confers sensitivity to fludioxonil, although the *S. cerevsiaie* is naturally resistant to the compound because it lacks an endogenous group III HHK [11]. Therefore, fludioxonil kills fungi in a group III HHK-dependent manner whether the encoding gene is expressed endogenously and heterologously.

We sought to exploit the fact that HHKs render fungi exquisitely sensitive to drugs that target this signaling pathway to identify candidate compounds with broad and potent antifungal activity. We harnessed a Hik1-expressing strain of *S. cerevisiae* as a cell-based reporter to develop a high throughput screen for compounds with group III HHK-dependent activity. After screening compound libraries, we identified two novel compounds that exerted significant activity across multiple genera of human fungal pathogens, including mold, yeast, and drug-resistant patient isolates. Analysis revealed that these compounds do not act directly on HHKs. However, microarray analysis provided insight into their modes of action and these compounds exhibit promising features as strong leads for drug development including robust, fungicidal activity against *in vitro* and *in vivo C. albicans* biofilm and synergy with fluconazole.

## Materials and Methods

### Fungal strains and growth conditions

The fungal strains used in this study were mostly human patient isolates and are listed in Table S1. In addition to *C. albicans* and *A. fumigatus*, they included non-albicans *Candida* spp., *Cryptococcus* spp., *Rhizopus oryzae* and *Fusarium solani*. The *Saccharomyces cerevisiae* reporter strain heterologously expresses Hik1, a group III HHK from *Magnaporthe grisea*, under the regulation of the galactose promoter, *gal1*, and was generously provided by Takayuki Motoyama from RIKEN (Wako, Japan). *S. cerevisiae* was incubated at 30°C. Complete medium was yeast peptone dextrose (YPD), and the minimal medium was yeast synthetic complete (SC) [12]. *Candida* spp. cultures were maintained on YPD at 30°C. *Cryptococcus* spp., *Aspergillus* spp., *R. oryzae*, and *F. solani* were grown on YPD at 37°C.

### High-throughput screen for small molecules

**Figure 1:**
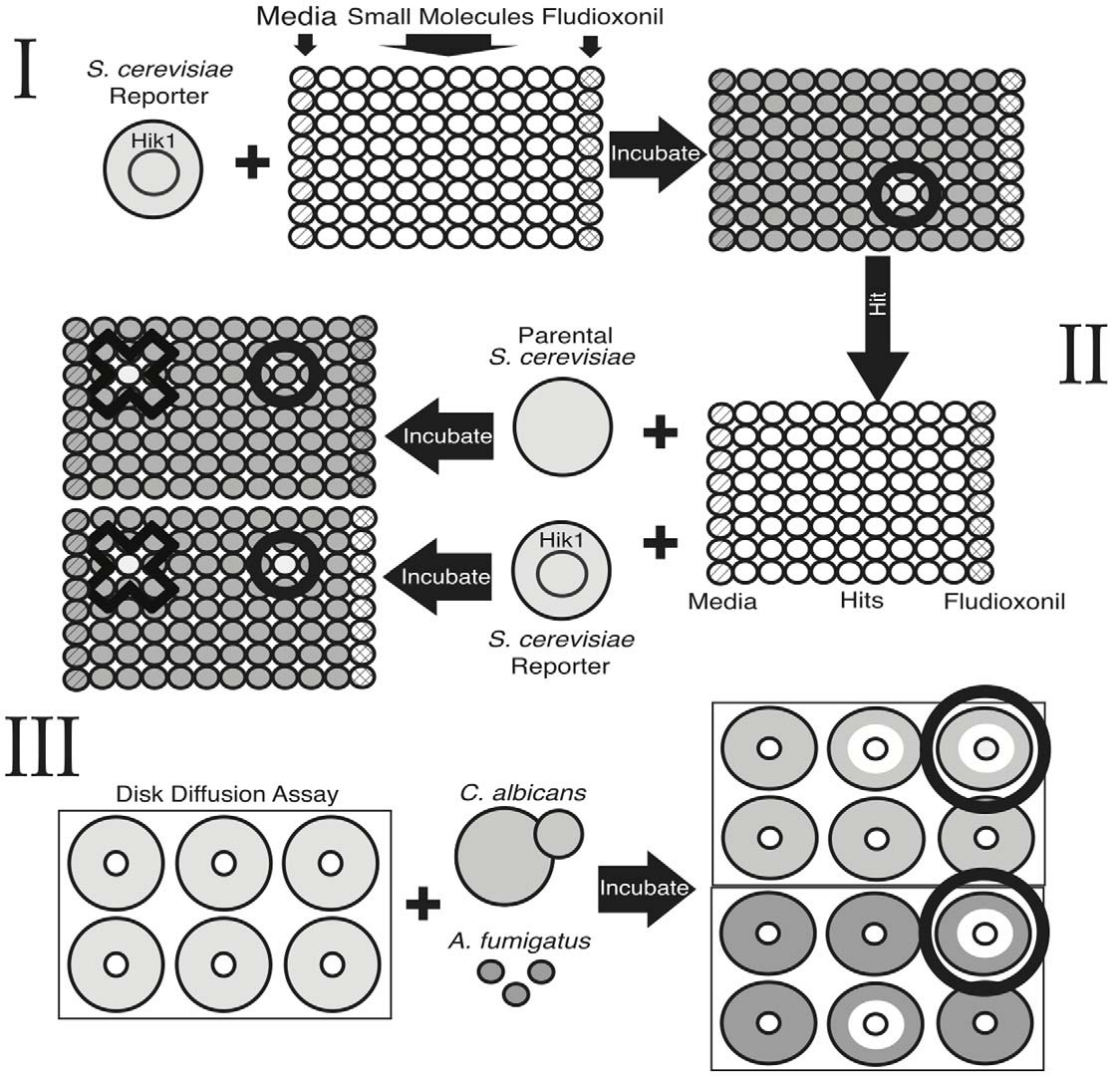
Hik1 *Saccharomyces* bioassay small molecule screen schematic. The small molecule screen was performed in three steps. (I) The Hik1-expressing *Saccharomyces* strain was seeded into 96-well plates containing 10 µM of small molecules. Media and fludioxonil containing wells served as negative and positive controls respectively. Hit compounds were defined as molecules that inhibited growth by at least 50% after overnight incubation at 30°C (black circle). (II) Hit compounds were assayed against parental and Hik1-expressing *Saccharomyces* to identify only molecules with Hik1-dependent activity. Compounds that inhibited the growth of both strains were discarded (black crosses). Molecules that inhibited Hik1-Saccharomyces growth by at least 50%, but did not hinder growth of the parental strain by more than 10% were considered hits (black circle). (III) Activity against the fungal pathogens *C. albicans* and *A. fumigatus* was assessed by disc diffusion. Top agar was seeded with yeast or spores, compound containing discs were then placed on the solidified agar, and the plates were incubated overnight at 37°C. Compounds that generated zones of inhibition (white circle) against both organisms were subjected to further analysis.

The small molecule screen was performed in three stages (Figure 1). In the primary screen, the *S. cerevisiae* strain expressing Hik1 under the control of a galactose-inducible promoter was seeded at 0.1 OD600 nm in 96-well plates containing SC media that lacked uracil and contained galactose. Small molecules (Maybridge Chemical Company; NIH Clinical Collection; Prestwick Chemical) were screened at a concentration of 10 µM. Wells containing media and fludioxonil (Sigma) served as negative and positive controls, respectively. The plate was incubated at 30°C overnight and growth was quantified by measuring OD600 nm with a plate reader. Compounds that caused a growth reduction >50%, relative to medium control were subjected to a second screen.

In the secondary screen to determine if the growth inhibition was Hik1-dependent, compounds were re-tested against the parental and Hik1-expressing *Saccharomyces* strains. The Hik1-expressing strain was grown under the same conditions as above, while the parental strain was grown in SC plus uracil with glucose. Small molecules that reduced the growth of the Hik1-expresing strain by >50%, and the parental wild-type strain by <10% were considered hits.

### Antifungal disk diffusion assay

0.5% top agar was seeded at 1.5×10^4^ yeast/ml from an overnight culture of *C. albicans* grown in YPD medium. 600 µl of top agar *C. albicans* suspension was added to each well of a 6-well plate containing 5 ml of YPD solid media. After the top agar solidified, sterile 6-mm paper discs (Fisher) containing 10 µg of small molecule were placed in each well. The compounds were stable and kept as a powder until they were compounded for testing in assays. A DMSO (Sigma) containing disc was added to each plate as a negative control. The plates were incubated at 37°C for 24 hrs, and activity was assessed based on the size of the zone of inhibition. Disk diffusions with *A. fumigatus* were performed following the same protocol, with the only difference being that the top agar was seeded with 1×10^6^ conidia/ml.

### Measurement of drug minimal inhibitory concentration (MIC) against fungi

The MIC of compounds against *Candida* spp. and *Cryptococcus* spp. was determined following CLSI protocol M27-A3. Briefly, overnight liquid cultures of *Candida* spp. or *Cryptococcus* spp. grown in YPD were enumerated using a hemocytometer, suspended in RPMI (Sigma) buffered with MOPS (Sigma) to pH 7.0 (RPMI/MOPS) to a density of 3×10^3^ per ml, and 100 µl of yeast was added to 96-well plates containing media and drug titrations in triplicate. The plates were incubated overnight at 37°C and the MIC was defined as the lowest concentration that prevented visible growth. The reported MICs are representative of at least two independent experiments. The MIC of compounds against *A. fumigatus*, *R. oryzae*, and *F. solani* was determined following CLSI protocol M38-A2, which is identical to M27-A3 protocol except that the seeding inoculum was at 2×10^4^ spores/ml. The commercial antifungal drugs fluconazole, voriconazole, and amphotericin B were purchased from the pharmacy of the University of Wisconsin Hospital and Clinics, Madison, WI.

The fungicidal or fungistatic activity of each compound against the isolates was determined as follows: A 100 µl aliquot was removed from the well representing the MIC of the compound, and spread onto a YPD plate. Wells containing isolates exposed to media alone or DMSO were plated as controls. The plates were incubated at 37°C for 48 hours, and compounds that prevented visible growth were considered fungicidal.

### RNA isolation from *C. albicans* after compound 13 or 33 exposure

A time course of *C. albicans* exposure to compounds was performed as follows. Briefly, an overnight culture of *C. albicans* grown in YPD at 37°C was diluted to 0.1 OD600 and grown at 37°C to exponential phase (0.4–0.5 OD600). After the incubation period, compounds were added at concentrations that caused a 50% reduction in CFU after a 3.5-hour incubation compared to DMSO (0.75 µg/ml for compound 13 and 3 µg/ml for compound 33). Samples were collected at 0, 20, 40 and 60 minutes. The cells were pelleted, supernatant was removed, and the cell pellet was flash frozen with liquid nitrogen. Samples were stored at −80°C until processed for RNA isolation.

RNA was extracted following the yeast RNA extraction protocol from the RNeasy Mini/Maxi Handbook (Qiagen). After RNA isolation, the sample was applied to a RNeasy Maxi Spin column and DNase (Ambion) treated according to the kit protocol. The RNA quality and integrity was verified with an Agilent 2100 Bioanalyzer (Agilent Technologies).

### Microarray analysis

cDNA labeling and microarray hybridizations were performed as described [13]. Briefly, dye-swapped duplicate hybridizations were performed on biological replicates for each time point (t = 0, 20, 40, 60 min) compared to cells treated with DMSO for 60 min. Briefly, 20 µg of total RNA was reverse transcribed using 9 ng of oligo(dT)_21_ in the presence of Cy3 or Cy5-dCTP (Invitrogen) and 400 U of Superscript III reverse transcriptase (Invitrogen). After cDNA synthesis, template RNA was degraded by adding 2.5 units RNase H (Promega) and 1 µg RNase A (Pharmacia) followed by incubation for 15 min at 37°C. The labeled cDNAs were purified with QIAquick PCR Purification Kit (Qiagen). Prior to hybridization Cy3/Cy5-labeled cDNA was quantified using a NanoDrop ND-1000 UV-VIS spectrophotometer (NanoDrop) to confirm dye incorporation. The labelled cDNA was hybridized to microarrays spotted with 6037 70mer oligonucleotide probes (GEO Platform GSE25822). Fluorescence intensity data was analyzed in Genespring Gx version 7.3 (Agilent Technologies) and MultiExperiment Viewer 4.7 (http://www.tm4.org/mev/). To account for changes in transcriptional profiles that occurred between t = 0 and t = 60, the drug-treated fluorescence ratios for each gene at t = 20, 40 and 60 min were divided by their ratios at t = 0. Data was reduced by selecting 1180 genes with a 2-fold change in transcript abundance under at least two conditions and visualized by hierarchical clustering.

Gene set enrightment analysis (GSEA) [14] was performed on the t = 20 min data using the GSEA Preranked tool and the weighted enrichment statistics on 6387 gene sets each containing 5–500 genes. Statistical significance was estimated from 1000 permutations. Enrichment maps were constructed with Cytoscape 2.8 [15] and the Enrichment Map 1.1 plug-in using the default settings.

### Heavy Metal Stress

Flasks containing YPD were seeded with 1500 yeast/mL from a 6-hr culture of *C. albicans* grown at 30°C shaking at 250 rpm. CdCl2 was added at concentrations from 0.004–0.031 mM in two-fold dilutions. After overnight incubation shaking at 30°C, 96-well round bottom plates were seeded with 50 µl of Cd or medium-exposed yeast at 1500 yeast/mL. Each pretreatment group was exposed to 50 µl YPD containing compound 33 (0.125–0.500 µg/ml), solvent control, or medium alone in triplicate. After incubation at 30°C overnight, growth was assessed using the XTT assay and growth reduction was determined relative to medium control.

### Hemolytic assay of compound toxicity

Compound hemolytic activity was assessed as described [16]. Briefly, freshly obtained human red blood cells (RBC) were washed three times with PBS and centrifuged at 2500 rpm for five minutes. A suspension of 1% RBC in PBS was added to 96-well plate containing two-fold dilutions of drugs ranging from 300–0.3 µg/ml in triplicate. PBS and 1% Triton X-100 (Sigma) served as negative and positive controls. After a 1-hour incubation at 37°C, the plate was centrifuged at 1500 rpm for five minutes and 50 µl of supernatant was transferred to a fresh 96-well plate. Hemoglobin release was quantified by measuring OD405 nm and values of the compound-only control plate were subtracted to remove any drug absorbance. The OD of cells exposed to 1% Triton X-100 represented 100% lysis; the OD of cells incubated in PBS represented O%.

### *C. albicans in vitro* biofilm assay

*C. albicans* biofilm sensitivity to drug was assessed using an *in vitro* assay as described [17]. Briefly, RPMI (Sigma), buffered to pH 7.0 with MOPS (Sigma), was seeded with 1×10^6^ yeast/ml from an overnight *C. albicans* (SC5314) grown in YPD at 30°C. The interior wells of flat-bottom 96-well plates were seeded with 100 µl of the yeast suspension. The exterior wells were filled with 200 µl PBS. The plate was wrapped in parafilm and foil and incubated at 37°C for 6 hours. The plate was gently washed twice with PBS. One column of wells was filled with media to serve as a growth control. The remaining columns were seeded in triplicate with compound, fluconazole, or amphotericin B in two-fold dilutions ranging from 0.1–25 µg/ml. After overnight incubation at 37°C, plates were gently washed twice with PBS. The tetrazolium dye, XTT (sodium 2,3,-bis(2-methoxy-4-nitro-5-sulfophenyl)-5-[(phenylamino)-carbonyl]-2H-tetrazolium inner salt) (Sigma) was used to assay the viability of cells [18]. Growth reduction was determined relative to the medium control based on OD490 nm.

The *in vitro* biofilm assay was also used to measure compound synergy with fluconazole as described [19]. After incubation to allow biofilm formation, compound 13 or 33 (0.4–25 µg/ml) and fluconazole (62.5–1000 µg/ml) were added to wells alone and in combination to 96-well plate. The XTT assay was used to determine growth reduction of drug-treated wells relative to media-treated wells. Synergy was quantified using the fractional inhibition concentration (FIC) that resulted in 25% growth reduction. FIC was calculated using the formula: [(EC25 of drug A in combination/EC25 drug A alone)+(EC25 of drug B in combination/EC25 of drug B alone)]. Values ≤0.5 indicate synergy.

### *In vivo* rat denture model of a *C. albicans* biofilm

The *in vivo* efficacy of compounds was assayed using a *C. albicans* biofilm denture model as described [20]. Briefly, specific-pathogen-free male Sprague-Dawley rats (Harlan Laboratories) were anesthetized and immunosuppressed with a single dose of cortisone (200 mg/kg subcutaneously) one day prior to infection. Dentures were then placed in the animals [20]. After the denture material had solidified, the dentures were inoculated with 100 µl of a 1×10^8^ cells/ml *C. albicans* solution. *C. albicans* biofilms were topically treated with compounds 13 (20 µg/ml), 33 (40 µg/ml), or saline control once per day. After 48 hours of treatment, the animals were sacrificed and the devices were removed. The dentures were placed in 2 ml 0.15 M NaCl, sonicated for ten minutes and vortexed. The viable burden of *C. albicans* was quantified by measuring colony-forming units (CFU) on agar at 30°C. Serial dilutions (1∶10) were plated on Sabouraud dextrose agar (Sigma) containing 2.5 µg/ml chloramphenicol and incubated at 30°C.

### Statistical analysis

The effect of pretreatment with heavy metal on the sensitivity of *C. albicans* to compound 33 was analyzed by two-way ANOVA. The burden of *C. albicans* in rat denture biofilm and the results of *C. albicans* biofilm sensitivity to compounds were assessed via an unpaired t-test. All statistical evaluations were performed using Graphpad Prism software (Version 5.0d for Mac OS X). P values of <0.05 were considered significant.

### Ethics Statement

The University of Wisconsin Animal Care and Use Committee approved all work with animals in this study. The Institutional Review Board approved collection of peripheral blood from volunteer donors who provided appropriate written consent.

## Results

### Identification of compounds 13 and 33 using a Hik-1 reporter bioassay

We performed a high-throughput, target-based screen to identify compounds that act in an HHK-dependent manner against the Hik1-expressing *Saccharomyces* reporter strain (Figure 1). In the primary screen, 19,762 small molecules and the positive control fludioxonil were assayed for activity against the reporter strain. Fludioxonil and 314 (1.6%) of the small molecules inhibited *S. cerevisiae* growth by more than 50% (Table 1). In the secondary screen to assess whether the small molecules acted in a Hik1-dependent manner, 57 of the small molecules inhibited the growth of the reporter strain by more than 50%, and the parental *S. cerevisiae* strain by less than 10% (Table 1). Thus, 57 compounds were carried forward as candidates for further testing against pathogenic fungi, using disk diffusion assays.

**Table 1:** Hik1 *Saccharomyces cerevisiae* small molecule screen results.

Screen	# Compounds tested	# Hits
Primary Screen	19,762†	314
Secondary Screen	314	57
Disk Diffusion Screen	40	15

† 18,432 compounds from Maybridge HitFinder Collection; 1,150 compounds from NIH Clinical Collection; 880 compounds from Prestwick Chemical Library.

**Figure 2:**
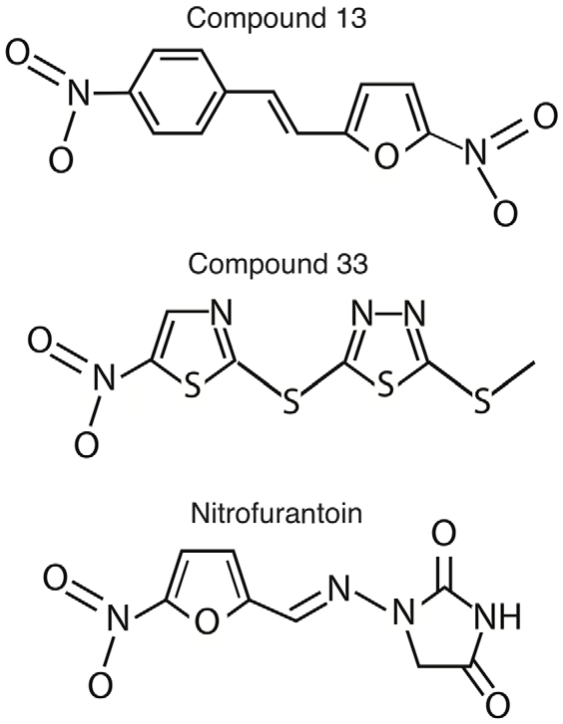
Small molecule structures. The chemical names of compounds 13 and 33 are 2-nitro-5-(4-nitroStyryl)furan (MW = 260.20) and 2-(methylthio)-5-[(5-nitro-1,3-thiazol-2-yl)thio]-1,3,4-thiadiazole (MW = 292.39) respectively.

Robust activity against *C. albicans* and *A. fumigatus* is a high priority for antifungal drug leads, since these are the two most prevalent and medically significant, systemic fungal pathogens. Disk diffusion experiments revealed that 15 of the small molecules generated visible zones of inhibition against both fungi (Table 1). Of these 15 candidates, compounds 13 and 33 produced the largest zones of inhibition against these fungi (data not shown), and they became the focus of our further studies. Compounds 13 and 33 are heterocyclic compounds containing nitro group(s), which is a structure similar to the commercial antimicrobial nitrofurantoin (Figure 2).

### Compounds 13 and 33 show potent, broad-spectrum antifungal activity

Because agar disk diffusion assays are only qualitative measures of activity, we performed quantitative assays. Using broth microdilution assays, compounds 13 and 33 were highly active against fungal pathogens spanning multiple genera, including pathogenic molds (Table 2). The MICs of compounds 13 and 33 against a collection of 20 isolates of *A. fumigatus* were 0.39 µg/ml and 0.39–0.78 µg/ml, respectively, which was in the range of those measured for voriconazole (0.20–1.56) and amphotericin B (0.20–1.56) (Table 2). Compound 33 yielded an MIC of 0.39 µg/ml against *Fusarium solani*, which was similar in potency to the activity measured for amphotericin B (Table 2). *Rhizopus oryzae* isolates were highly sensitive to compound 13 (MIC 0.2­0–0.39 µg/ml) and 33 (MIC 0.78–1.56 µg/ml), and also to amphotericin B (MIC 0.20 µg/ml) (Table 2).

**Table 2:** Spectrum of activity of compounds 13 and 33 against pathogenic fungi.*

Organism	13	33	Fluconazole	Voriconazole	Amphotericin B
*Candida albicans* wild type (4)	0.25–0.50	0.25–0.50	0.50–0.78	NT+	0.13–0.25
*Candida albicans* drug-resistant (3)	0.25–0.50	0.25–0.50	>25	NT	0.13–0.25
*Candida glabrata* (5)	<0.20	3.12–6.24	3.13–>25	NT	0.78
*Candida krusei* (3)	6.24–12.5	0.78–1.56	>25	NT	1.56
*Candida lusitaniae* (3)	1.56–3.12	1.56–3.12	3.12–6.24	NT	<0.20
*Cryptococcus neoformans* var. *grubii* (1)	0.25	0.13	2	NT	0.06
*Cryptococcus gattii* (2)	0.39–0.78	0.78	>25	NT	0.78
*Aspergillus fumigatus* (20)	0.39	0.39–0.78	NT	0.20–1.56	0.20–1.56
*Fusarium solani* (1)	6.25	0.39	NT	NT	0.39
*Rhizopus oryzae* (2)	0.20–0.39	0.78–1.56	NT	NT	0.20

* Broth microdilution quantification of compound MIC (µg/ml) against yeast and filamentous fungal pathogens. The number of fungal strain isolates tested is in parentheses. The values are representative of at least two independent experiments.

+ NT = not tested.

Compounds 13 and 33 were also highly active against pathogenic yeast, showing similar potency to the conventional antifungals. We tested several pathogenic species of *Candida*. *C. albicans* exhibited respective MICs of 0.25–0.50, 0.25–0.50, 0.50–0.78, and 0.13–0.25 µg/ml for compounds 13 and 33, fluconazole, and amphotericin B (Table 2). In contrast to *C. albicans*, *Candida glabrata* showed high MIC values to compound 33, fluconazole, and amphotericin B (MIC of 3.12–6.24, 3.13–>25, and 0.78 µg/ml). However, all strains of *C. glabrata* were sensitive to compound 13 (MIC<0.25 µg/ml) (Table 2). The activity of compounds 13 and 33 against *C. lusitaniae* was similar to that of fluconazole, but less than that for amphotericin B, with MICs of 1.56–3.12, 1.56–3.12, 3.12–6.24, and <20 µg/ml respectively (Table 2).

Compounds 13 and 33 also had potent activity against *Cryptococcus* species. *Cryptococcus neoformans* was more susceptible to compounds 13 and 33 than to fluconazole, showing MICs of 0.25, 0.13, and 2 µg/ml respectively (Table 2). Although both isolates of *C. gattii* were resistant to fluconazole (MIC>25 µg/ml), compounds 13 and 33 showed significant activity that was comparable to amphotericin B with respective MICs of 0.39–0.78, 0.78, and 0.78 µg/ml (Table 2).

Compounds 13 and 33 were tested to determine if their activity was fungistatic or fungicidal. Both compounds exerted fungicidal activity against all of the fungal genera tested above, in the same concentration range as observed for growth inhibition.

### Compounds 13 and 33 show potent activity against fluconazole-resistant strains of *C. albicans*

The emergence of *C. albicans* strains that are resistant to the frontline drug fluconazole is a significant therapeutic problem [21]. Fluconazole-resistant *C. albicans* isolates (MIC>25 µg/ml) were highly sensitive to compounds 13 and 33, showing MIC values of 0.25–0.50 µg/ml, which was similar to the values for other *C. albicans* isolates (Table 2). Moreover, *C. krusei*, which is naturally resistant to fluconazole (MIC values>25 µg/ml), was as sensitive to compound 33 as amphotericin B, yielding MIC values of 0.78–1.56 µg/ml and 1.56 µg/ml respectively (Table 2).

### Direct activity of compounds 13 and 33 against HHKs in pathogenic fungi

Deletion of group III HHK renders numerous fungi resistant to fludioxonil [22]–[25]. To see if the activity of compounds 13 and 33 required HHKs in pathogenic fungi, we assessed the sensitivity of *A. nidulans* wild type and group III HHK (NikA) knockout strains to compounds 13 and 33 and fludioxonil (Table 3). Deletion of NikA engendered resistance to fludioxonil. Wild type *A. nidulans* had an MIC of 0.63 µg/ml and the ΔNikA strain, an MIC>25 µg/ml (Table 3). In contrast, deletion of NikA had no effect on the sensitivity of *A. nidulans* to compounds 13 or 33. Wild type and ΔNikA strains had MICs of 3.13 µg/ml for both compounds (Table 3). The thirteen other hits from the small molecule screen also lacked group III HHK-dependent activity in *A. nidulans* (data not shown).

**Table 3:** Compounds 13 and 33 are not Group III HHK-dependent in *Aspergillus nidulans* *.

Strain	Compound 13	Compound 33	Fludioxonil
Wild Type	3.13	3.13	0.63
ΔNikA	3.13	3.13	>25

* Broth microdilution quantification of compound MIC (µg/ml) against parental and ΔNikA strains of *A. nidulans*. The values are representative of three independent experiments.

### Microarray analysis of *C. albicans* to discern compound modes of action

**Figure 3:**
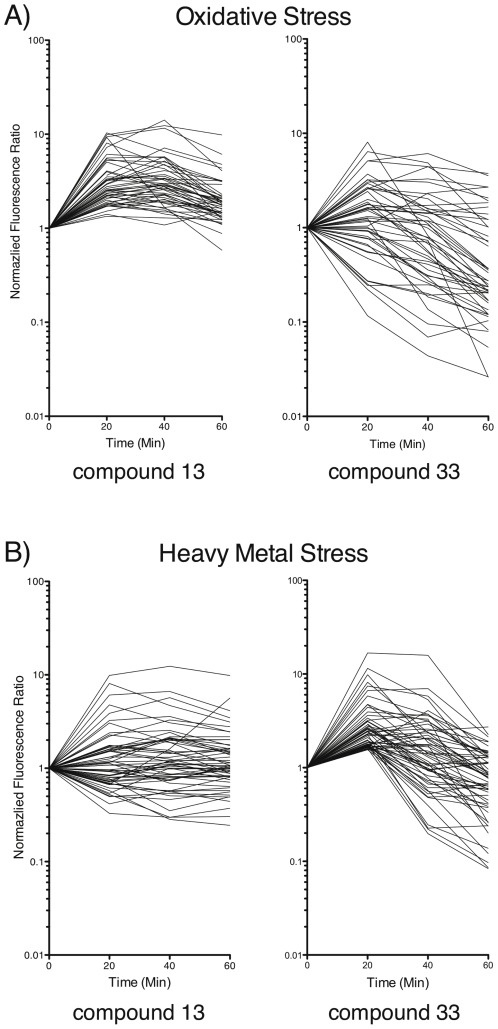
Microarray analysis of *C. albicans* exposed to compounds 13 and 33. Changes in transcript expression of oxidative (A) and heavy metal (B) stress response gene clusters during a time course of *C. albicans* exposed to compounds 13 or 33. The fluorescence intensity (i.e. transcript abundance) relative to the given transcript value at time 0 is shown for *C. albicans* exposed to compound for 20, 40 and 60 minutes.

We sought further insight into the mode of action of our compounds since they exhibited such potent and broad-spectrum activity against fungi. Transcripts of *C. albicans* were analyzed over the course of compound exposure for 60 minutes (http://www.ncbi.nlm.nih.gov/geo/query/acc.cgi?token=bvmtzccmsecqgbi&acc=GSE35105). By 40 minutes of exposure, 285 transcripts were upregulated and 362 transcripts were downregulated >2-fold in response to compound 13. The respective numbers for compound 33 were 364 transcripts upregulated and 476 transcripts down-regulated >2-fold (see File S1). Although some of the transcripts appeared similarly modulated following the addition of either compound (Figure S1), we did not observe a significant correlation between genes that were upregulated with both compounds. In contrast, the downregulated genes were mostly associated with GO terms such as Transport, Carbohydrate and Lipid Metabolic Processes, Response to Stress and Response to Drug. Comparison of the transcript profiles with other profiles and annotations by Gene Set Enrichment Analysis (GSEA) [14] revealed that *C. albicans* exposed to compound 13 significantly up-regulated 48/127 transcripts associated with oxidative stress [26] (Figure 3A) (Table S2) (File S2). Other gene sets enriched among those upregulated by compound 13 were involved in oxygen and reactive oxygen metabolic processing, antioxidant activity, and oxidative stress (data not shown).

Exposure to compound 33 also lead to a unique signature of gene expression. Sixty of the 107 genes associated with the heavy metal stress response gene cluster [26] were upregulated upon incubation with compound 33 (Figure 3B) (Table S3). The known heavy metal stress-induced genes Cpr6, Sba1, and Sis1 were upregulated 6.65, 5.84, and 4.74 fold, respectively [26], [27]. Therefore, oxidative damage appears to be involved in the mode of action of compound 13, while compound 33 induces heavy metal stress.

### Fungi deficient in DNA damage repair have increased sensitivity to compound 13

Oxidative stress commonly results in DNA damage [28]. To determine if DNA damage is involved in the action of compound 13 or 33, we assessed their activity against DNA repair-deficient strains of *C. albicans* and *A. nidulans* (Table 4). The DNA repair-deficient *C. albicans* were much more sensitive than the parental strain to compound 13, with MICs of 0.08 µg/ml and 0.63 µg/ml respectively (Table 4). The inability to repair DNA damage also increased the sensitivity of *A. nidulans* to compound 13. The mutant strain had an MIC of 0.16 µg/ml compared to an MIC of 0.63 µg/ml for the wild type. The inability to repair DNA damage did not affect the sensitivity of either fungus to compound 33, with all strains showing an MIC of 1.25 µg/ml (Table 4). Nor did DNA repair-deficient fungi exhibit altered sensitivity to amphotericin B (Table 4). These findings are compatible with a mode of action mediated by compound 13 (but not 33) that involves oxidative stress.

**Table 4:** DNA repair-deficient fungi have an increased sensitivity to compound 13 but not 33.*

Strain	Compound 13	Compound 33	Amphotericin B
*C. albicans* wild type	0.63	1.25	0.63
ΔRad2	0.08	1.25	0.63
ΔRad10	0.08	1.25	0.63
*A. nidulans* wild type	0.63	1.25	20
ΔuvsJ1	0.16	1.25	>20

* The MIC (µg/ml) of compounds 13 and 33 and amphotericin B against *C. albicans* and *A nidulans* strains was determined using the assay. These data are representative of three independent experiments.

#### Pre-exposure to heavy metal enhances sensitivity to compound 33

**Figure 4:**
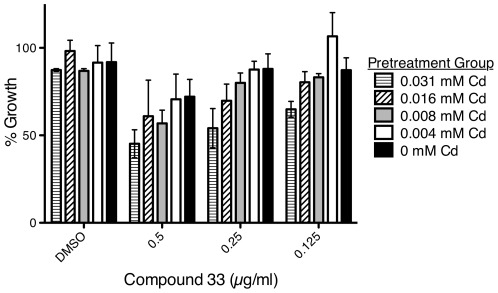
Pretreatment with heavy metal stress increases the sensitivity of *Candida albicans* to compound 33. *C. albicans* cultures were incubated overnight at 30°C in the presence of cadmium concentrations shown. After exposure to this heavy metal, *C. albicans* was exposed to compound 33 at concentrations shown and incubated at 30°C overnight. Growth was assessed using the XTT assay and growth reduction was measured relative to the medium control. The data are the mean ± SD of at least three samples per treatment group and are representative of three independent experiments. There was a significant interaction for the two conditions (F[12, 60] = 2.59, p = 0.008; ANOVA test) indicating that pre-exposure of *C. albicans* to heavy metal increased sensitivity to compound 33.

We hypothesized that pre-exposure to heavy metal would alter the sensitivity of *C. albicans* to compound 33. Pre-exposure of *C. albicans* to osmotic stress has been shown to alter its resistance to subsequent exposure to the osmotic stress-inducing peptide histatin 5 [29]. We found that pre-exposing *C. albicans* to a heavy metal stress increased its sensitivity to compound 33 (Figure 4). Pre-exposure of *C. albicans* to cadmium in sub-lethal amounts significantly enhanced sensitivity of *C. albicans* to compound 33 in a concentration-dependent manner. Heavy metal stress therefore may contribute to the mechanism of action of compound 33.

### Compound toxicity

**Figure 5:**
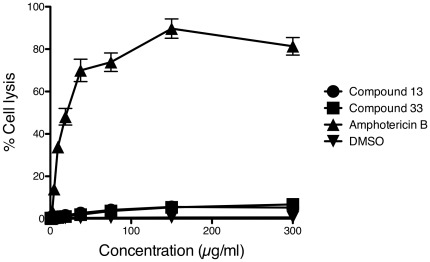
Toxicity of compounds 13 and 33. The hemolytic activity of compounds 13, 33, and amphotericin B was measured (OD405 nm) by the release of hemoglobin from red blood cells after a one-hour exposure to compound at 37°C. 0.1% Triton X-100 was used as a control. The data are represented as the mean ± SD of three samples per treatment group from three independent experiments.

Induction of DNA damage or oxidative stress might suggest possible compound toxicity. We performed a standard assay of cell membrane fragility assay to explore the toxicity of compounds 13 and 33 (Figure 5). Amphotericin B, a commonly used antifungal drug known to damage cell membranes, lysed RBCs at drug concentrations as low as 20 µg/ml, whereas compounds 13 and 33 failed to lyse these cells even at concentrations of 300 µg/ml, which is well in excess of their MICs against fungi (Figure 5).

### Activity of compounds 13 and 33 against drug-resistant *C. albicans* biofilm

**Figure 6:**
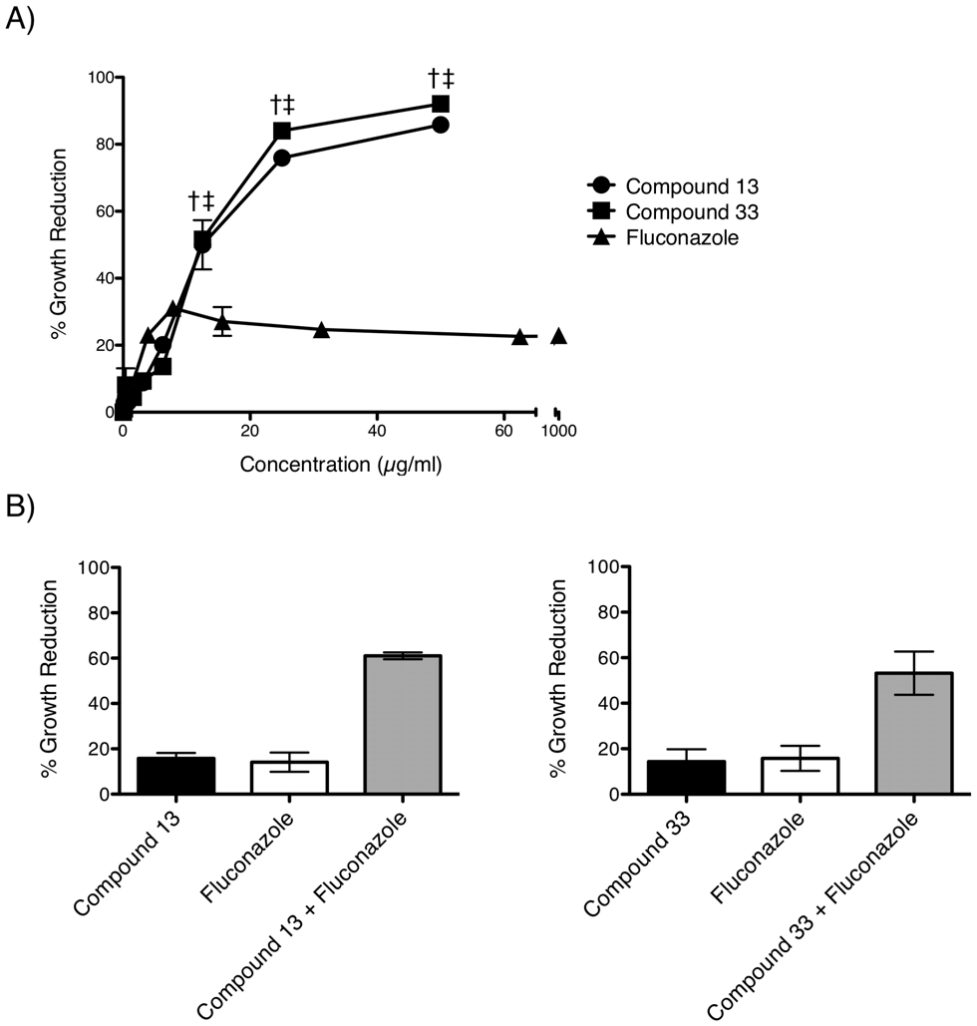
Activity of compounds 13 and 33 against *C. albicans* biofilms *in vitro*. (A) Six-hour *in vitro C. albicans* biofilms were incubated overnight at 30°C in the presence of compound 13 or 33 (1.56–50 µg/ml) or fluconazole (62.5–500 µg/ml) as a control. After drug exposure, *C. albicans* biofilm viability was assessed using XTT and growth reduction was determined relative to medium control biofilm. An unpaired t-test revealed that *C. albicans* biofilm was significantly more sensitive to compounds 13 (†) and 33 (‡) than to fluconazole (1000 µg/ml) (p = <0.05). The data are the mean ± SD of three samples per treatment group, and are representative of two independent experiments. (B) Synergistic activity of compounds with fluconazole in *C. albicans* biofilm *in vitro*. Six-hour *in vitro C. albicans* biofilms were incubated overnight at 30°C with compound 13 (1.6 µg/ml) or 33 (6.3 µg/ml) and fluconazole (1000 µg/ml) alone and in combination. After drug exposure, the XTT assay was used to assess growth reduction of the drug-treated wells relative to media control. The data are representative of a trend of synergy observed over a range of concentrations of compounds 13 and 33 (0.4–25 µg/ml) with fluconazole (62.5–1000 µg/ml), and are displayed as the mean ± SD of two independent experiments.

*C. albicans* forms biofilms on implanted medical devices. These biofilms are resistant to the front-line drug fluconazole and represent life-threatening bloodstream infections [30]. We assayed activity of compounds 13 and 33 against *C. albicans* biofilm using an *in vitro* assay (Figure 6A). Compounds 13 and 33 were more active than fluconazole against *C.albicans* biofilms. Both compounds reduced biofilm growth >50% at concentrations as low as 12.5 µg/ml, whereas fluconazole failed to inhibit *C. albicans* growth by >30% even at elevated drug concentrations of 1000 µg/ml. Thus, compounds 13 and 33 each appeared to be highly active against *C. albicans* biofilms.

### Compounds 13 and 33 exert synergy with fluconazole against *C. albicans* biofilm

In view of the clinical utility of fluconazole, we explored whether compounds 13 or 33 might act synergistically with fluconazole against *C. albicans* biofilms. *In vitro C. albicans* biofilm indeed was hypersensitive to a combination of 13 or 33 with fluconazole (Figure 6B). The FIC indices of compounds 13 or 33 with fluconazole were 0.26 and 0.32, respectively, which indicate a significant synergistic interaction of both compounds with fluconazole (Table 5).

**Table 5:** Compounds 13 and 33 exert synergy with fluconazole against *in vitro Candida albicans* biofilm*.

Compound	EC25	EC25 with Fluconazole	FIC
13	6.30±0.00	1.20±0.57	0.26±0.09
33	4.71±2.25	1.20±0.57	0.32±0.00

* FIC index = [EC25(A in combination)]/[EC25(A alone)]+[EC25(B in combination)]/[EC25(B alone)], where A is compound 13 or 33 and B is fluconazole. An index of <0.5 indicates synergism. The results are averages ± SD of two independent experiments.

### Compounds 13 and 33 are active in vivo against *C. albicans* biofilm

**Figure 7:**
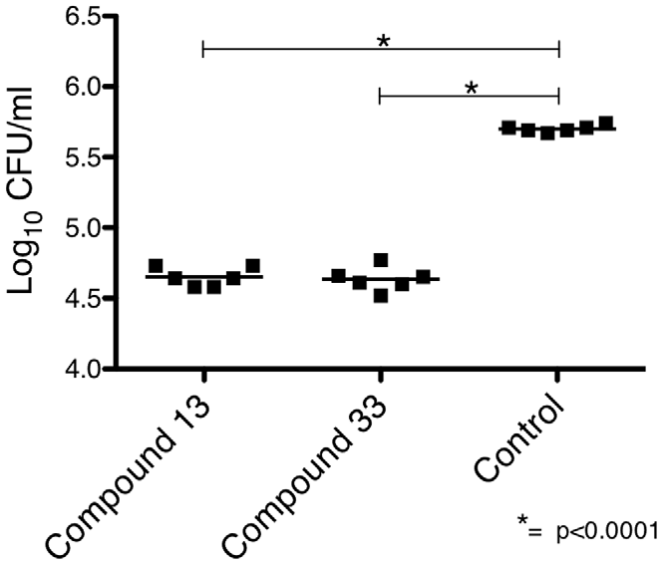
Activity of compounds 13 and 33 against *C. albicans* biofilm *in vivo*. *C. albicans* biofilms established using a rat denture model were exposed to compounds 13 (20 µg/ml), 33 (40 µg/ml) or saline once per day. After 48 hours of treatment, the dentures were removed, sonicated, and serial dilutions plated to determine the fungal burden as measured by CFU. Each symbol represents CFU of an individual rat denture, with the line indicating the treatment group mean. The data are representative of three independent experiments.

In view of the significant activity of the compounds against *C. albicans* biofilm *in vitro*, we evaluated their activity *in vivo* against *C. albicans* biofilm. In initial studies, we ascertained that both compounds exhibited significant protein binding (data not shown), making serum levels negligible following systemic administration, and treatment of systemic infection inconsistent. For this reason, to minimize the confounding effects of protein binding, we studied the activity of compounds 13 and 33 following topical application in a rat denture model of *C. albicans* biofilm infection (Figure 7). Compounds 13 and 33 each reduced the fungal burden of dentures by more than one order of magnitude, when compared to animals that received control treatment.

## Discussion

Novel antifungal drugs are badly needed to combat the increasing clinical challenge posed by fungal pathogens. We utilized a *S. cerevisiae* reporter to create a high throughput bioassay to identify novel antifungal compounds small molecule libraries. Disk diffusion assays of initial hits revealed compounds 13 and 33 as two antifungal drug leads with potent and potentially broad spectrum activity against two major fungal pathogens *A. fumigatus* and *C. albicans*. In further testing using broth microdilution assays, compounds 13 and 33 yielded MICs against these two fungi, other molds and *C. neoformans* with similar potency to currently used antifungals such as fluconazole and amphotericin B. Importantly, both compounds showed fungicidal activity against mold and yeast.

While compounds 13 and 33 exert potent, broad-spectrum activity, they do not appear to act directly against the group III HHKs – the mode of action that was sought in this screen. Why did our screen not identify compounds that directly act on group III HHKs? There may be several explanations. First, we screened only a limited number of small molecules (∼20,000). Though sufficient for a preliminary screen, large-scale screening efforts often include hundreds of thousands of compounds. Second, *S. cerevisiae has* only one HHK, Sln1, while *A. nidulans* encodes 11 HHKs [31]. As a result, HHKs redundant in function to NikA could complement its deletion explaining retained compound activity against the ΔNikA strain. Third, it is possible that Hik1 overexpression “reprograms” *S. cerevisiae* to become sensitive to compounds 13 and 33. The activity of compounds 13 and 33 therefore requires Hik1 in *S. cerevisiae*, but the compounds do not directly target Hik1.

Microarray analysis revealed that compound 13 induced the up-regulation of genes associated with an oxidative stress response. The GSEA network analysis also showed a concordant increase in gene families associated with an oxidative stress response. Oxidative stress is known to damage DNA, and we found that DNA repair-deficient fungi were much more sensitive to compound 13 than were wild type strains. Together, these data argue that compound 13 acts in a manner dependent on the induction of an oxidative stress response.

*C. albicans* exposed to compound 33 induced transcripts associated with the stress response to heavy metals. We also found that pre-exposing *C. albicans* to heavy metal stress, by initial growth in cadmium, increased its sensitivity to compound 33. Although this result supports possible involvement of heavy metal stress in the mode of action of compound 33, the finding was unanticipated since prior work showed that pre-exposure to a given stress engenders resistance [29]. How do we explain this discrepancy? Vylokova *et al.* [29] examined osmotic stress while we studied heavy metal stress [29]. The different stressors could explain the discrepancy. It is also possible that residual cadmium associated with *C. albicans* after pretreatment resulted in the exposure of yeast simultaneously to heavy metal and compound 33. Similarly, Vylokova *et al.* [29] observed that concurrent incubation of *C. albicans* with osmotic stress together with the osmotic stress-inducing peptide histatin 5 significantly enhanced its sensitivity.

The enhanced sensitivity of DNA repair-deficient fungi to compound 13 is notable from a drug development standpoint, since it suggests the compound may damage DNA. Nevertheless, there are FDA approved antibiotics that damage DNA as part their mode of action. Nitrofurantoin, a commonly used antimicrobial, damages DNA as part of its action [32]. Compound 13 contains a nitro group structurally, like in nitrofurantoin, where it is reduced to generate oxidative stress [33]. Although compound 33 also contains nitro groups, it did not induce oxidative stress response genes, nor did compound 33 have increased activity against DNA repair-deficient fungi. Thus, the nitro group of compound 13, but not 33, is likely reduced leading to oxidative stress and DNA damage, a possible toxicity concern.

Toxicity is a concern with any potential therapeutic. Highly toxic compounds should have been eliminated during the small molecule secondary screen, when compounds that inhibited the growth of wild type *S. cerevisiae* were removed. We also used a standard cell membrane lysis assay to assess compound toxicity. Amphotericin B lysed RBCs at low concentrations, whereas compounds 13 or 33 had little effect even at the high concentrations of 300 µg/ml. There are many different types of cell and animal toxicity assays and we limited our initial analysis *in vitro* to this standard assay, but other cell-based testing may be desirable. We also found that while compounds 13 and 33 bound serum protein substantially, making systemic administration challenging, mice tolerated substantial doses with no overt toxicity (data not shown). Thus, from the *in vitro* studies in wild-type *S. cerevisiae*, and the studies with human RBCs, and in mice, we conclude that compounds 13 and 33 are not general cytotoxins.

Fungal biofilms represent one of the most challenging types of fungal infections facing patients and physicians. Compounds 13 and 33 had robust activity against *C. albicans* biofilm. Compounds 13 and 33 also were highly active *in vivo* in a rat denture model of *C. albicans* biofilm infection. Whereas fluconazole alone had little activity against *in vitro* biofilm, compounds 13 and 33 synergized significantly with fluconazole. Fluconazole-induced oxidative stress may have enhanced its synergy with compounds 13 and 33.

The azole antifungals inhibit lanosterol 14 α–demethylase, a crucial enzyme in the biosynthesis of ergosterol [34], but recent studies have suggested that the generation of oxidative stress may also be involved in their mechanism of action. For example, the addition of free radical scavengers rendered *C. albicans* resistant to the azole antifungal miconazole [35]. Furthermore, incubation with sub-inhibitory concentrations of fluconazole caused *C. albicans* to upregulate oxidative stress response genes and made the organism more resistant to killing by phagocytes [36]. Exposure to sub-inhibitory concentrations of fluconazole had a similar affect on *C. neoformans* [37]. Oxidative stress has also been postulated to be involved in the synergy of compounds with the azoles. For example, the synergistic activity against *C. albicans* of fluconazole with polyphenol curcumin I, a plant-derived antifungal, was abolished by the addition of an antioxidant [38]. Thus, the mechanism of synergy between fluconazole and compounds 13 and 33 likely involved oxidative stress.

In summary, by employing a simple high-throughput *S. cerevisiae* bioassay, we discovered two compounds - 13 and 33 - with potent fungicidal activity against yeast and molds across multiple genera that frequently infect human patients. The compounds also work against fungal biofilms and act in synergy with conventional antifungal drugs. Compounds 13 and 33 therefore represent potentially valuable antifungal drug leads.

## Supporting Information

Figure S1 **Hierarchical clustering of transcriptional profiles.** Transcriptional profiles from 1080 genes with a change in transcript abundance (see File S1) of at least 2-fold were clustered according to their Euclidean Distance. Upregulated genes are colored in yellow while downregulated genes are colored in blue. *C. albicans* was exposed to the compounds for 20, 40 and 60 minutes as described in Methods.
(TIFF)

Table S1 **Fungal strains used in this study.**
(DOC)

Table S2 **Cluster of oxidative stress response genes identified by microarray analysis.**
(DOC)

Table S3 **Cluster of heavy metal stress response genes identified by microarray analysis.**
(DOC)

File S1 **Transcriptional Profiles.** The first worksheet contains the averaged and normalized fluorescence ratios for 1080 genes whose transcripts were modulated 2-fold in at least two conditions. The second worksheet contains the complete dataset from each individual hybridization.
(XLS)

File S2 **GSEA results.** Results of a gene set enrichment analysis (GSEA) [12] performed on the t = 20 min compound 13 and compound 33 data using the Preranked tool and the weighted enrichment statistics on 6387 gene sets each containing 5–500 genes. Statistical significance was estimated from 1000 permutations. Table lists enriched gene sets where the p-value is smalller or equal to 0.05 while the FDR is smaller or equal to 25%. A description of the GSEA statistics is provided at http://www.broadinstitute.org/gsea/doc/GSEAUserGuideFrame.html?_Interpreting_GSEA_Results. Each gene sets includes a suffix to indicate whether it originates from transcriptional profiles (_UP and _DN), GO Terms (_BIO, _CEL, _MOL), signaling pathways as defined by the Candida Genome Database (_PATH), transcription factor binding motifs (_MOTIF), the *S. cerevisiae* BIND protein-protein interaction database (_BIND), or *S. cerevisiae* genetic interaction groups (_SGA).
(XLS)
