# Supplementary figures and images for: Identification and Characterization of Antifungal Compounds Using a Saccharomyces cerevisiae Reporter Bioassay
Source: PLoS One. 2012 May 4;7(5):e36021. doi: 10.1371/journal.pone.0036021 (PMC3344848; doi:10.1371/journal.pone.0036021)

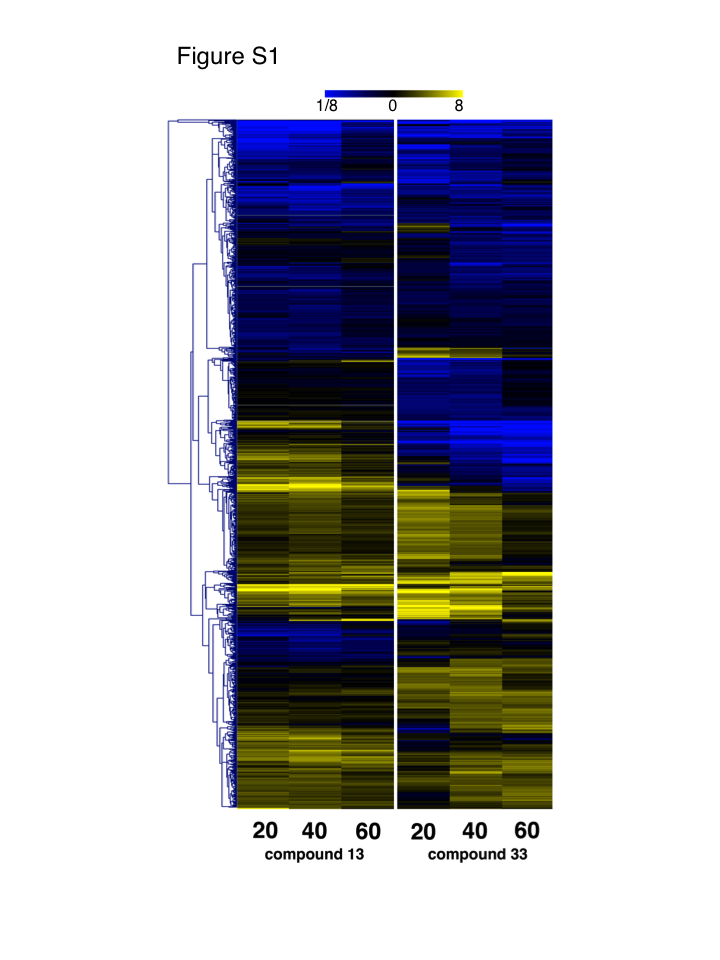

Supplement: Figure S1 — Hierarchical clustering of transcriptional profiles. Transcriptional profiles from 1080 genes with a change in transcript abundance (see File S1) of at least 2-fold were clustered according to their Euclidean Distance. Upregulated genes are colored in yellow while downregulated genes are colored in blue. C. albicans was exposed to the compounds for 20, 40 and 60 minutes as described in Methods. (TIFF) [file pone.0036021.s001.tiff]
