# Supplementary material for: Identification and Characterization of Antifungal Compounds Using a Saccharomyces cerevisiae Reporter Bioassay
Source: PLoS One. 2012 May 4;7(5):e36021. doi: 10.1371/journal.pone.0036021 (PMC3344848; doi:10.1371/journal.pone.0036021)
Supplement: Table S1 — Fungal strains used in this study. (DOC) [file pone.0036021.s002.doc]

**Table S1: Fungal strains used in this study**

| **Organism** | **Notes** | **Reference or Source** |
| --- | --- | --- |
| *Saccharomyces cerevisiae* |  |  |
| W303-1A | Parental; ATCC 208352 | ATCC |
| Hik1 Expressing | Hik1 under galactose promoter | Motoyama et al., 2005 |
| *Candida albicans* |  |  |
| SC5314 | Clinical Isolate | Gillum et al., 1984 |
| 18804 |  | ATCC |
| DKaC39 |  | Legrand et al., 2008 |
| 412 | Clinical Isolate | Andes et al., 2006 |
| 1002 | Clinical Isolate | Andes et al., 2006 |
| 2-76 | Clinical Isolate | Andes et al., 2006 |
| 2823 | Fluconazole-Resistant Clinical Isolate | Andes et al., 2006 |
| 2307 | Fluconazole-Resistant Clinical Isolate | Andes et al., 2006 |
| 1299 | Fluconazole-Resistant Clinical Isolate | Andes et al., 2006 |
| *Candida glabrata* |  |  |
| 1906 | Clinical Isolate | This Study |
| 1907 | Clinical Isolate | This Study |
| 1-215-8377 | Clinical Isolate | This Study |
| 1-218-8640 | Clinical Isolate | This Study |
| 3-218-8073 | Clinical Isolate | This Study |
| *Candida krusei* |  |  |
| 1685 | Clinical Isolate | This Study |
| 3459 | Clinical Isolate | This Study |
| 3-219-8544 | Clinical Isolate | This Study |
| *Candida lusitaniae* |  |  |
| 556 | Clinical Isolate | This Study |
| 3459 | Clinical Isolate | This Study |
| 3595 | Clinical Isolate | This Study |
| *Cryptococcus neoformans* var*. grubii* |  |  |
| H99 | Serotype A | ATCC |
| *Cryptococcus gattii* |  |  |
| 1266 | Clinical Isolate | Personal Communication* |
| 1269 | Clinical Isolate | Personal Communication* |
| *Aspergillus fumigatus* |  |  |
| Af293 |  | Fungal Genetics Stock Cntr |
| PI 1-20 | Clinical Isolate | This Study^†^ |
| *Fusarium solani* |  |  |
| M1565 | Clinical Isolate | This Study^†^ |
| *Rhizopus oryzae* |  |  |
| 04335-8297 | Clinical Isolate | This Study^†^ |
| 08107-8036 | Clinical Isolate | This Study^†^ |

* Strain was kindly provided by Dee Carter from the University of Sydney, Australia

† Strain was obtained from the University of Wisconsin Hospital and Clinic Clinical Microbiology Lab
