# Supplementary material for: Identification and Characterization of Antifungal Compounds Using a Saccharomyces cerevisiae Reporter Bioassay
Source: PLoS One. 2012 May 4;7(5):e36021. doi: 10.1371/journal.pone.0036021 (PMC3344848; doi:10.1371/journal.pone.0036021)
Supplement: Table S2 — Cluster of oxidative stress response genes identified by microarray analysis. (DOC) [file pone.0036021.s003.doc]

**Table S2: Cluster of oxidative stress response genes identified by microarray analysis***

| **Systemic** | **Compound 13** | **Compound 33** | **Common** |
| --- | --- | --- | --- |
| orf19.6229 | 10.34 | 1.31 | CAT1 |
| orf19.3131 | 9.87 | 1.79 | OYE32 |
| orf19.5877 | 9.41 | 5.11 | ATF1 |
| orf19.7585 | 9.26 | 0.27 | INO1 |
| orf19.7085 | 8.08 | 2.69 |  |
| orf19.113 | 7.25 | 0.98 | CIP1 |
| orf19.4653 | 6.12 | 0.27 |  |
| orf19.3443 | 5.60 | 0.93 | OYE2 |
| orf19.2285 | 5.48 | 0.12 |  |
| orf19.847 | 5.28 | 2.00 | YIM1 |
| orf19.2165 | 5.06 | 0.66 |  |
| orf19.3122 | 4.08 | 0.22 | ARR3 |
| orf19.4290 | 4.01 | 1.01 | TRR1 |
| orf19.93 | 3.65 | 5.11 |  |
| orf19.6548 | 3.53 | 0.81 | ISU1 |
| orf19.2262 | 3.35 | 1.62 |  |
| orf19.6066 | 3.28 | 3.04 |  |
| orf19.1149 | 3.26 | 0.93 | MRF1 |
| orf19.3121 | 3.24 | 8.07 | GST1 |
| orf19.2446 | 3.19 | 1.64 |  |
| orf19.1340 | 2.97 | 1.87 |  |
| orf19.5094 | 2.93 | 1.27 | BUL1 |
| orf19.3150 | 2.77 | 0.76 | GRE2 |
| orf19.2396 | 2.65 | 0.92 | IFR2 |
| orf19.4177 | 2.56 | 3.21 | HIS5 |
| orf19.4337 | 2.54 | 0.24 |  |
| orf19.6272 | 2.53 | 2.94 |  |
| orf19.3639 | 2.53 | 0.47 |  |
| orf19.86 | 2.46 | 1.52 |  |
| orf19.6059 | 2.36 | 1.76 | TTR1 |
| orf19.4307 | 2.32 | 3.23 |  |
| orf19.5258 | 2.24 | 0.55 |  |
| orf19.1623 | 2.24 | 0.56 | CAP1 |
| orf19.238 | 2.21 | 1.16 | CCP1 |
| orf19.1107 | 2.16 | 1.57 |  |
| orf19.5870 | 2.07 | 1.04 | CTP1 |
| orf19.3537 | 1.99 | 0.25 |  |
| orf19.2461 | 1.89 | 0.77 | PRN4 |
| orf19.2990 | 1.84 | 2.58 | XOG1 |
| orf19.2989 | 1.80 | 6.39 |  |
| orf19.6947 | 1.78 | 2.59 | GTT11 |
| orf19.2693 | 1.74 | 2.42 | GST2 |
| orf19.2467 | 1.73 | 1.24 | PRN1 |
| orf19.1406 | 1.71 | 2.60 |  |
| orf19.3115 | 1.71 | 1.59 |  |
| orf19.5259 | 1.52 | 0.64 |  |
| orf19.5337 | 1.41 | 3.70 | UBC15 |
| orf19.7611 | 1.34 | 1.20 | TRX1 |

* Expression of the oxidative stress response gene cluster after 20 minute exposure to compounds 13 and 33. The raw microarray datasets can be viewed via GEO: <http://www.ncbi.nlm.nih.gov/geo/query/acc.cgi?token=bvmtzccmsecqgbi&acc=GSE35105>
