# Supplementary material for: Identification and Characterization of Antifungal Compounds Using a Saccharomyces cerevisiae Reporter Bioassay
Source: PLoS One. 2012 May 4;7(5):e36021. doi: 10.1371/journal.pone.0036021 (PMC3344848; doi:10.1371/journal.pone.0036021)
Supplement: Table S3 — Cluster of heavy metal stress response genes identified by microarray analysis. (DOC) [file pone.0036021.s004.doc]

**Table S3: Cluster of heavy metal stress response genes identified by microarray analysis***

| **Systemic** | **Compound 13** | **Compound 33** | **Common** |
| --- | --- | --- | --- |
| orf19.2344 | 0.84 | 16.77 | ASR1 |
| orf19.882 | 0.54 | 11.49 | HSP78 |
| orf19.6398 | 1.68 | 9.84 |  |
| orf19.7602 | 0.57 | 8.17 |  |
| orf19.822 | 1.15 | 7.43 |  |
| orf19.7654 | 0.71 | 6.65 | CPR6 |
| orf19.5749 | 0.80 | 5.83 | SBA1 |
| orf19.3861 | 1.32 | 4.74 | SIS1 |
| orf19.6073 | 0.75 | 4.71 | HMX1 |
| orf19.4246 | 1.51 | 4.41 |  |
| orf19.3226 | 1.16 | 3.93 |  |
| orf19.5645 | 0.73 | 3.62 | MET15 |
| orf19.3433 | 1.55 | 3.39 | OYE23 |
| orf19.675 | 0.61 | 3.20 |  |
| orf19.2092 | 0.96 | 3.14 |  |
| orf19.4076 | 0.52 | 3.11 | MET10 |
| orf19.6117 | 1.59 | 2.93 |  |
| orf19.6402 | 0.50 | 2.87 | CYS3 |
| orf19.7350 | 0.80 | 2.76 |  |
| orf19.7085 | 0.69 | 2.69 |  |
| orf19.6763 | 0.72 | 2.63 | SLK19 |
| orf19.6520 | 0.92 | 2.61 |  |
| orf19.6408 | 9.86 | 2.57 |  |
| orf19.3160 | 0.42 | 2.47 | HSP12 |
| orf19.3795 | 1.71 | 2.44 | AGP3 |
| orf19.5180 | 6.08 | 2.43 | PRX1 |
| orf19.2693 | 0.33 | 2.42 | GST2 |
| orf19.1153 | 0.68 | 2.30 | GAD1 |
| orf19.4099 | 0.67 | 2.28 | ECM17 |
| orf19.5312 | 0.50 | 2.26 |  |
| orf19.682 | 0.69 | 2.19 |  |
| orf19.5280 | 0.84 | 2.10 | MUP1 |
| orf19.2738 | 1.74 | 1.98 | SUL2 |
| orf19.251 | 1.20 | 1.95 |  |
| orf19.717 | 1.08 | 1.88 | HSP60 |
| orf19.2737 | 0.96 | 1.84 |  |
| orf19.3131 | 3.05 | 1.79 | OYE32 |
| orf19.988 | 0.93 | 1.79 |  |
| orf19.7297 | 1.08 | 1.78 |  |
| orf19.2613 | 8.08 | 1.78 | ECM4 |
| orf19.1159 | 1.13 | 1.76 |  |
| orf19.5673 | 1.52 | 1.75 | OPT7 |
| orf19.7150 |  | 1.74 | NRG1 |
| orf19.946 | 0.95 | 1.73 | MET14 |
| orf19.1559 | 1.61 | 1.72 | HOM2 |
| orf19.5811 | 1.55 | 1.72 | MET1 |
| orf19.1167 | 1.61 | 1.71 |  |
| orf19.6404 | 0.77 | 1.69 | GSH2 |

*Expression of the oxidative stress response gene cluster after 20 minute exposure to compounds 13 and 33. The raw microarray datasets can be viewed via GEO: <http://www.ncbi.nlm.nih.gov/geo/query/acc.cgi?token=bvmtzccmsecqgbi&acc=GSE35105>
